# Bark and Ambrosia Beetles Show Different Invasion Patterns in the USA

**DOI:** 10.1371/journal.pone.0158519

**Published:** 2016-07-26

**Authors:** Davide Rassati, Massimo Faccoli, Robert A. Haack, Robert J. Rabaglia, Edoardo Petrucco Toffolo, Andrea Battisti, Lorenzo Marini

**Affiliations:** 1 Department of Agronomy, Food, Natural Resources, Animals, & Environment (DAFNAE), University of Padua, Legnaro (PD), Italy; 2 USDA Forest Service, Northern Research Station, Lansing, Michigan, United States of America; 3 USDA Forest Service, Forest Health Protection, Washington, D. C., United States of America; University of Innsbruck, AUSTRIA

## Abstract

Non-native bark and ambrosia beetles represent a threat to forests worldwide. Their invasion patterns are, however, still unclear. Here we investigated first, if the spread of non-native bark and ambrosia beetles is a gradual or a discontinuous process; second, which are the main correlates of their community structure; third, whether those correlates correspond to those of native species. We used data on species distribution of non-native and native scolytines in the continental 48 USA states. These data were analyzed through a beta-diversity index, partitioned into species richness differences and species replacement, using Mantel correlograms and non-metric multidimensional scaling (NMDS) ordination for identifying spatial patterns, and regression on distance matrices to test the association of climate (temperature, rainfall), forest (cover area, composition), geographical (distance), and human-related (import) variables with β-diversity components. For both non-native bark and ambrosia beetles, β-diversity was mainly composed of species richness difference than species replacement. For non-native bark beetles, a discontinuous invasion process composed of long distance jumps or multiple introduction events was apparent. Species richness differences were primarily correlated with differences in import values while temperature was the main correlate of species replacement. For non-native ambrosia beetles, a more continuous invasion process was apparent, with the pool of non-native species arriving in the coastal areas that tended to be filtered as they spread to interior portions of the continental USA. Species richness differences were mainly correlated with differences in rainfall among states, while rainfall and temperature were the main correlates of species replacement. Our study suggests that the different ecology of bark and ambrosia beetles influences their invasion process in new environments. The lower dependency that bark beetles have on climate allowed them to potentially colonize more areas within the USA, while non-native ambrosia beetles, being dependent on rainfall, are typically filtered by the environment.

## Introduction

International movement of non-native forest insects represents a severe threat to forests worldwide [1,2]. Bark and ambrosia beetles (Coleoptera: Curculionidae, Scolytinae) represent one of the most successful groups of invaders, in part because they are easily transported in wood products and packaging materials [3,4]. Continuing increases in international trade are expected to pose even greater risks of invasion by these organisms [5–10]. For these reasons, improved understanding of the possible drivers of the invasion process of bark and ambrosia beetles is recognized as a key research priority.

Although many factors influencing current geographic distribution and species richness of non-native species have been already examined [8,10], drivers and mechanisms of assembly of non-native communities are poorly studied. In this context, analyses of β-diversity may provide insights into these mechanisms [11–14], although whether this approach can identify casual processes is still under debate [15–20]. Spatial β-diversity can be generally defined as the extent of change in community composition, or degree of community differentiation, between different locations [21]. This differentiation can be partitioned into different components, each representing distinct ecological processes: species richness difference, species replacement, and nestedness. Several indices have been proposed to quantify the individual components [22–24], which have generated some confusion given their ambiguous ecological interpretation [25]. A recently developed analytical framework partitions β-diversity into its species richness and replacement components [23]. This framework can be effectively used to unveil potential drivers of community assembly and understand the invasion process, but to date no studies investigating β-diversity patterns of non-native bark and ambrosia beetles have been produced. Such information would be valuable to managers interested in surveillance and control measures at points of entry or other high-risk sites.

Scolytines are generally divided into two main guilds according to their feeding habit [26]: bark beetles and ambrosia beetles. Bark beetles build their galleries primarily in the phloem or, less frequently, in the sapwood from which they take most nutrients [26]. They are characterized by relatively high host specificity, given their reliance on specific phloem characteristics, tree chemistry, and nutritional quality [26]. Many ambrosia beetles are instead relatively generalists in the host plants they colonize, feeding primarily on symbiotic fungi that they inoculate on the tunnel walls they bore in wood [26]. These different feeding habits have already been demonstrated to influence patterns of species richness of non-native scolytines in the USA: non-native ambrosia beetles, requiring suitable climatic conditions for the growth of their symbiotic fungi, are more numerous in wetter and warmer states, whereas non-native bark beetles, being less dependent on climate, are more homogenously distributed [8]. It is still unknown, however, whether the different feeding habits of scolytines also affect the assembly of their communities. For non-native bark beetles, given their high host specificity, considerable differences in species composition (i.e. high species replacement) could be expected among USA states that differ in forest composition; for non-native ambrosia beetles, instead, given their low host specificity, it could be predicted that only slight differences in species composition (i.e. low species replacement) among USA states exist, and that the latter may be more related to temperature or rainfall differences rather than to differences in forest composition.

The primary points of entry of non-native species are likely to be airports and seaports, which import goods from all over the world [4,27–29]. After the initial introduction, invaders must overcome a continuum of abiotic and biotic filters in order to successfully establish and spread in a new region [30]. Spread is affected by multiple variables [30] and not all non-native species that arrive at a given point of entry are able to immediately occupy the new environment, as confirmed by the higher number of non-native species established in coastal areas compared with interior continental areas [8,27,31]. Community assembly of non-native bark and ambrosia beetles should follow the rule of the distance decay of similarity, where the similarity between two locations often decreases as the geographical distance between them increases [32]. This is usually due to a combination of at least two, not mutually exclusive, mechanisms: dispersal limitation and environmental filtering [11].

Dispersal limitation implies that differences in species dispersal capabilities lead to differences in community composition. However, multiple-site introductions and human-assisted dispersal [33–37] can increase the homogenization of non-native species communities. To test for these hypotheses, we analyzed spatial autocorrelation of species richness differences and species replacement separately for non-native bark and ambrosia beetles across the continental USA. The shape of the spatial autocorrelation could help to understand whether their spread is a gradual process mainly due to the natural dispersal of the beetles (e.g. positive significant autocorrelation of species replacement at short distance classes), a discontinuous process characterized by jump dispersal ahead of the established core population mainly due to human-assisted dispersal (e.g. lack of spatial trend in species richness differences) [38], or a combination of these two processes. Given that both bark and ambrosia beetles can fly long distances [39] and are commonly moved by humans via retail wood [40], wood chips [41], and packaging materials [27], it could be expected that both processes can affect their spread.

Environmental filtering assumes that species are able to reach all environmentally suitable sites, therefore environmental divergence determines differences in species richness and species replacement between different sites [42]. It could be predicted, for example, that the pool of non-native scolytines arriving in coastal areas are filtered by the environment as they spread into interior continental areas according to their feeding habit. Changes in community composition would therefore reflect species-specific niche differences in adaptive responses that have evolved along environmental gradients. Considering the relatively higher host specificity of bark beetles, we would expect that their spread would be limited primarily by the presence of their host [43]. In contrast, we expect that ambrosia beetles would be less constrained by host availability but could spread within the new environment wherever the climate allows development of the beetle and its symbiotic fungi [8]. To test for these hypotheses, we applied regression on distance matrices to understand the direction and the shape of the correlation of climate, forest, and human-related variables with both species richness differences and species replacement.

Furthermore, we compared non-native with native scolytine responses to climate, forest, geographical, and human-related variables. As non-native species share the habitat with the more abundant native species, it becomes interesting to simultaneously compare the processes shaping their community assembly.

## Materials and Methods

### Data on scolytine distribution

We used data from the continental 48 contiguous USA states. For each state, we had detailed information about the presence/absence of both non-native and native bark and ambrosia beetles. For native species the distributional data were gathered mainly from the monograph written by Wood [26], for non-native species we first used information from Wood [26,44], Wood and Bright [45], Bright and Skidmore [46,47], Haack [4,27], Rabaglia et al. [48], Cognato et al. [49], and Haack and Rabaglia [50], and then we integrated them with published and unpublished records obtained from the USDA Forest Service, Early Detection and Rapid Response (EDRR) project (Rabaglia et al. [51]). The latter project, which has been carried out since 2007, focused on detecting new populations of non-native bark and ambrosia beetles established in the USA. The project involves sampling 10–15 states every year, and, within each state, 7–10 sites (e.g. natural forests, parks, commercial areas, industrial areas) are selected and monitored using multi-funnel traps baited with generic lures [51]. Although the coarse spatial resolution of the data and the possible limitations that can affect their reliability (i.e. differences in trapping efforts among states), no information at a finer spatial resolution is currently available for this group of insects.

### Assigning scolytines to a feeding habit

We split the scolytines into three groups: bark beetles, ambrosia beetles, and seed-feeding beetles. In particular, we used definitions provided by Kirkendall et al. [52]: “bark beetles” include all the species breeding in inner bark (live and dead phloem tissues) or in the wood that use phloem cells or sapwood as the primary source of food, as well as pith and twig breeders; “ambrosia beetles” include all the species whose larvae feed primarily on symbiotic ambrosia fungi, which adult females cultivate in their tunnel systems in woody tissues; we did not distinguish among fungus-farming species that do and do not ingest wood as well as fungus; “seed-feeding beetles” include scolytines that breed in seeds and their encasing fruit tissues. We excluded from the analyses species that cannot be univocally associated to one of the three functional feeding groups (e.g. some *Hypothenemus* species which can develop both in twigs and seeds) or that develop on herbaceous plants and grasses (e.g. *Hylastinus obscurus* on legumes). In addition, we did not exclude bark beetle species that are known to have a close relationship with symbiotic fungi, such as *Dendroctonus*, *Ips*, *and Tomicus* spp. for two main reasons: a) the latter relationship is not an obligate one, although it can enhance larval and adult fitness [52], and b) the primary source of food is represented by the phloem [52]. For non-native species, the information needed to assign each bark and ambrosia beetle to the different functional groups were gathered from different sources (see S1 Table), whereas, for native species, we referred to Wood [26] and Kirkendall et al. [52]. We excluded the group of “seed-feeding beetles” from the analyses because it was represented by only a few species.

### Analyses of β-diversity

#### Species richness difference and species replacement

We used the method by Carvalho et al. [53] (see also Podani and Schmera [23] and Legendre [54]) to partition the compositional β-diversity into two components: species richness difference and species replacement. The general term β-diversity refers to the total compositional change between two communities irrespective of the process that originated each. The term “species richness difference” refers to the relative difference between the number of species that each site supports irrespective of any potential nestedness. In other words, it refers to the fact that one community may include a larger number of species than another, which may be due to various ecological processes [54]. The term “species replacement” indicates that one or more species present in one site are substituted by different species at another site. It refers to the well-known fact that species tend to replace each other along ecological gradients that are sufficiently long [54]. The two terms are therefore additive and can be generally defined as:

β-diversity=species replacement+species richness difference

The total compositional beta diversity between row cells is given by the Jaccard dissimilarity index:

$$\beta_{cc}=(b+c)/(a+b+c)$$

where “a” is the number of species common to both sampling units, b and c are the number of species exclusive to the first sampling unit and to the second sampling unit, respectively. β_cc_ is bounded between zero (perfect similarity) and one (maximum possible dissimilarity). This proportional measure can be partitioned into its replacement and richness difference component as given below [23,53].

The species richness difference between two sampling units is given by:

$$\beta_{rich}=│b-c│/(a+b+c)$$

The replacement component, i.e. the substitution of n species in a given sampling unit from n species in another site, is defined using the β_-3_ [55]:

$$\beta_{-3}=2\;*\;min(b-c)\;/\;(a+b+c)$$

where min(b,c) is the minimum number of exclusive species. This quantity is multiplied by 2 because each substitution involves two different species.

The indices were computed using the “vegdist” function in the package vegan version 3.2–5 [56] for R version 3.2.0 [57].

### Spatial autocorrelation

The pattern and significance of spatial autocorrelation across different geographical distance lags were examined using Mantel correlograms [58]. First, we created a geographical distance matrix between the 48 USA states (see below). Second, we divided the distance matrix into five distance classes using Sturge’s rule [59]. Mantel correlation coefficients were calculated for each distance class and tested for significance with a permutation test (n = 9999) based on a progressive Bonferroni correction (alpha = 0.05 [59]). As the Mantel correlogram was computed on a dissimilarity matrix (β-diversity), we coded the second distance matrix such that negative significant values of Mantel statistics corresponded to an increase in dissimilarity. On the other hand, positive significant values indicate that the communities are more similar than expected by chance (i.e. low beta-diversity). The response matrices were the replacement and species richness difference variables for bark and ambrosia beetles separately. All Mantel analyses were performed using the “mantel.correlog” function with default settings in the vegan package [56] implemented in R.

### Spatial patterns

To visualize the similarity patterns of non-native and native bark and ambrosia beetle communities of the USA states, we performed non-metric multidimensional scaling (NMDS) ordination using the function “metaMDS” (distance = “*jaccord”*, K = 2) in the vegan package [56]. We used NMDS because unlike methods which attempt to maximize the variance or correspondence between objects in an ordination, it attempts to represent − as closely as possible—the pairwise dissimilarity between objects in a low-dimensional space [59].

### Correlates

We included in the analyses several variables that can help explain species richness differences and species replacement among different USA states. For each USA state, these variables are indicative of the geographical distance, the volume of international trade (imports), and the environment (forest and climate).

#### Geographical distance

We measured the geographical distance (DIST) between each pair of USA states using the centroid projected in UTM WGS84 and then calculating a distance matrix between the sampling units using the “earth.dist” function in the fossil package version 0.3.7 [60] for R.

#### International trade

Wood packaging materials, such as pallets, inadvertently represent one of the most common means through which bark and ambrosia beetles are transported in international trade [29]. Total imports (IMP) are therefore expected to be more related to the potential introduction of non-native scolytines than simply imports of wood products (e.g., lumber). For this reason, we used the difference in log-transformed total value of imports between USA states as a proxy for the number of non-native scolytines. We acquired data on the average value of goods imported during the period 2008‒2010 from official economic statistics of the USA (Economic Census Bureau, US International Trade in Goods and Service FT900). We used data for the final destination of the imports rather than the first point of arrival because the vast majority of imports arrive in shipping containers that are not opened until they reach their final destination. See Marini et al. [8] for more details about the selection of this metric. We used the arithmetic difference between states to compute the distance matrix for import.

#### Environmental distances

Two distance matrices based on two climatic variables were used based on monthly data: mean annual temperature (TEMP) and mean annual precipitation (RAIN). Both were derived from the WorldClim database and corresponded to the bioclimatic variables BIO6 and BIO12 respectively ([61]; 1 km resolution;1960‒1990 period). Both variables were averaged within each USA state. Moreover, we included in the analyses a distance matrix based on forest vegetation differences (FOR) between states using the Bray-Curtis dissimilarity index. We retrieved current information on the area covered by 31 forest type categories from the USDA Forest Service, Forest Inventory Data Online (FIDO) [62]. Each category is defined as a physiognomically uniform group of plant associations sharing one or more dominant tree species. The FOR variable indicated whether two states are similar or different based on the number of forest types which are shared between them or are exclusive to either state. In addition, we considered the difference in extent (size) of area covered by forests per state, after the variable AREA was log-transformed to improve linearity and homogeneity of residual variance. All environmental matrices were calculated using the “vegdist” function in the vegan package [56] for R.

### Analyses of correlates of species richness difference and species replacement

We used multiple regression on distance matrices (MRM) [63] to test the overall direction and the shape of the association of the single environmental and geographical correlates on species richness differences (β_rich_) and species replacement (β_-3_). MRM performs regression between a response matrix and any number of explanatory matrices. Each explanatory matrix contained distances or similarities between all pairwise combinations of the 48 USA states of ecological and environmental factors, or other attributes such as imports. Tests of statistical significance were done by permutation [64]. MRM allows for the inclusion of quadratic terms in the model to account for non-linearity in the relationships. For each predictor we tested both linear and quadratic terms. All environmental and geographical correlate matrices were standardized using the function ‘stdize’ in the MuMIn package version 1.15.6 for R. The MRM analysis was carried out with the ‘MRM’ function in the ecodist package version 1.2.9 [65] for R.

Second, we used hierarchical partitioning (HP) [66] to evaluate the relative importance of explanatory distance matrices in explaining variation in species richness differences (β_rich_) and species replacement (β_-3_). HP jointly considers all possible models in a multiple regression and identifies the most likely causal factors. The analyses split the variation explained by each variable into a joint effect together with the other explanatory variables into an independent effect not shared with any other variable. HP was computed using the hier.part package version 1.0–3 [67] implemented in R. The estimated relative importance of each variable was represented by the size of its individual effect.

## Results

### Species richness difference and species replacement

A total of 480 scolytine species were analyzed for the continental USA, including 43 non-native and 437 native species. The number of non-native ambrosia beetles was higher than that of non-native bark beetles (25 and 18, respectively), whereas there were many more native bark beetles (387) than native ambrosia beetles (50). For both non-native bark beetles and non-native ambrosia beetles we found a mean value of β_rich_ greater than β_-3_ (0.30 vs. 0.16 and 0.50 vs 0.17, respectively), i.e. species richness differences contribute more than species replacement to differentiate non-native bark and ambrosia beetle communities at the scale of USA states. For both native bark and ambrosia beetles we found similar values between species richness difference and species replacement, with β_-3_ slightly higher than β_rich_ both for bark beetles (0.41 vs. 0.36) and ambrosia beetles (0.35 vs. 0.33).

### Spatial autocorrelation

**Fig 1:**
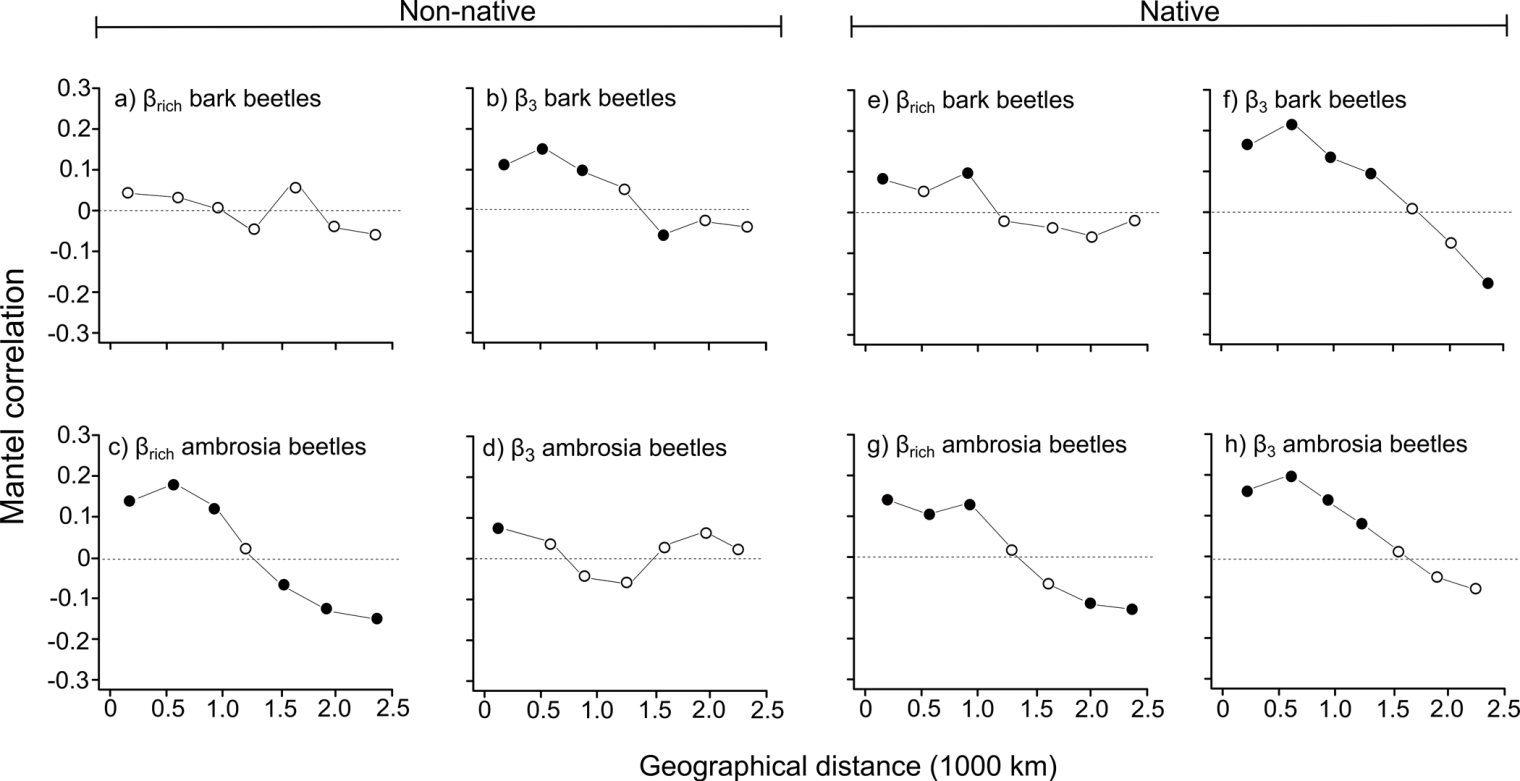
Mantel correlogram for non-native and native bark and ambrosia beetle dissimilarity using β_rich_ and β_-3_. Solid circles indicate significant positive or negative correlations (based on sequential Bonferroni corrections with α = 0.05) between compositional dissimilarity and geographical distance (based on longitude and latitude of centroid of the different USA states). Open circles indicate non-significant correlation.

When non-native bark beetle species were considered, the Mantel correlogram for β_rich_ showed no clear spatial trend (Fig 1A); for β_-3_, instead, a significant positive autocorrelation at the smallest lag distance classes, indicating that neighboring states had similar non-native bark beetle species composition, and a significant negative autocorrelation at medium distance class were apparent (Fig 1B). The Mantel correlogram computed for β_rich_ of non-native ambrosia beetle showed significant and positive autocorrelation at the smallest lag distance classes and a significant but negative autocorrelation at highest lag distance classes (Fig 1C), indicating that neighboring states had similar non-native ambrosia beetle species richness, whereas communities located in distant states were characterized by a different number of species. For the β_-3_ of non-native ambrosia beetle, only a significant and positive autocorrelation at the smallest distance class occurred (Fig 1D), indicating that neighboring states had a similar non-native bark beetle species composition whereas no clear trend was identified when the distance between states increased.

When native bark beetle species were evaluated, the Mantel correlogram for β_rich_ showed a significant and positive autocorrelation at small distance classes (Fig 1E), indicating that neighboring states had similar native bark beetle species richness, while no clear trend was identified when the distance between states increased. For β_-3_ of native bark beetles a significant and positive spatial autocorrelation at the smallest distance classes and a negative autocorrelation at highest distance class were present (Fig 1F), indicating that neighboring states had similar native bark beetle species composition, whereas communities located in distant states were composed by different species. The Mantel correlogram computed for β_rich_ of native ambrosia beetles showed significant and positive autocorrelation at the smallest lag distance classes and a significant but negative autocorrelation at highest lag distance classes (Fig 1G), indicating that neighboring states had similar native ambrosia beetle species richness, whereas communities located in distant states were characterized by different number of species. Instead, for the β_-3_ of native ambrosia beetles, a significant and positive autocorrelation at the smallest and intermediate distance classes were apparent (Fig 1H), indicating that neighboring states had similar native bark beetle species composition, whereas no clear trend was identified when the distance between them increased.

### Spatial patterns

**Fig 2:**
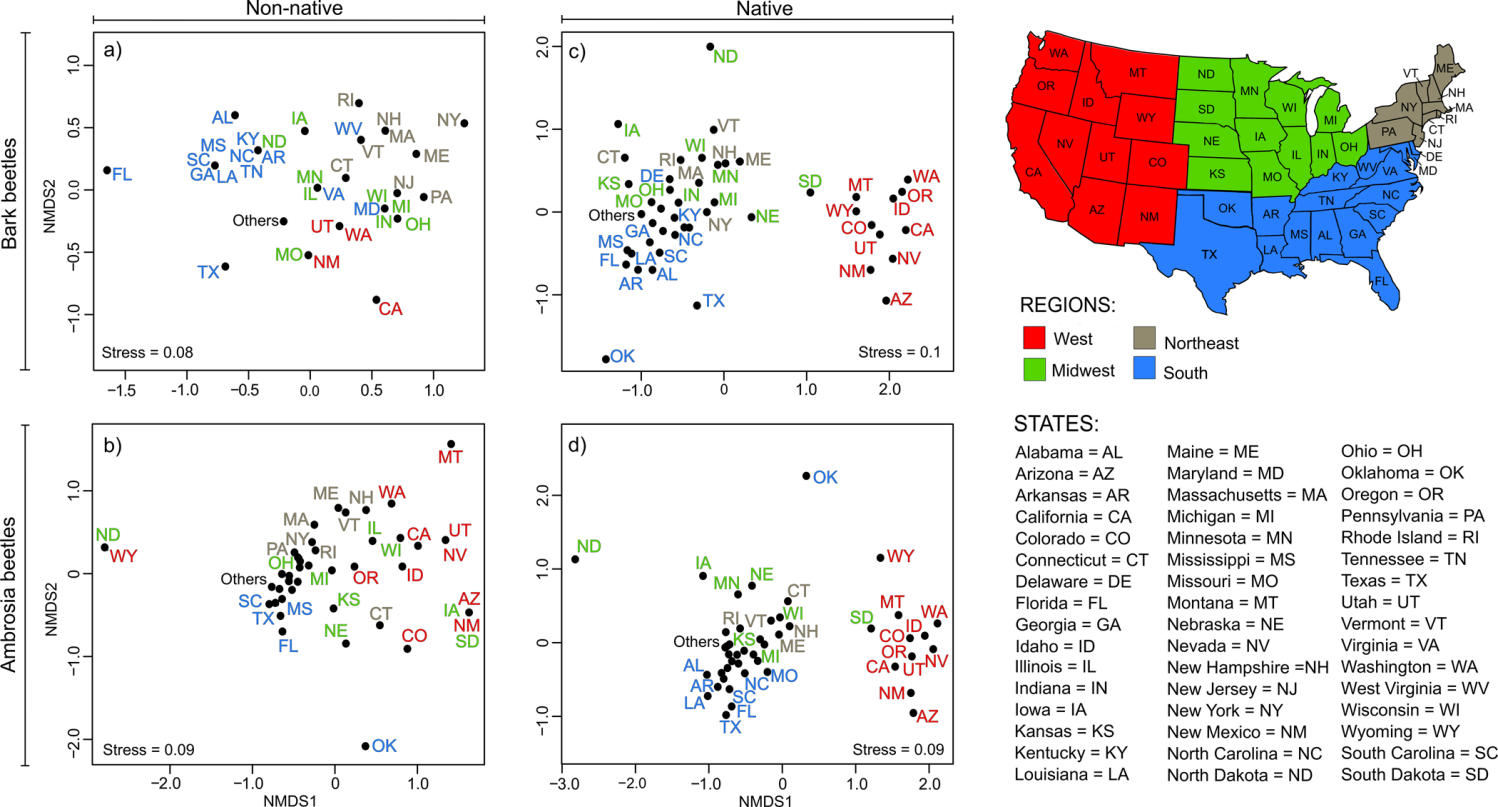
NMDS ordination plots based on Jaccard dissimilarities for non-native and native bark and ambrosia beetles communities of the 48 USA states (black dots). USA regions are distinguished following the Census Bureau definition; states are listed in alphabetical order. Given that non-native ambrosia beetles are not recorded in Minnesota, we removed the latter state from the analysis regarding this group of beetles.

For non-native bark beetles, the visual inspection of the NMDS ordination plot did not provide a clear spatial pattern of similarity, although a tendency of neighboring states to have similar or identical species communities (e.g. Mississippi, Georgia, South Carolina, and Louisiana in the South or New York, Maine, Massachusetts, and New Hampshire in the North East) is apparent (Fig 2A). For non-native ambrosia beetles, visual inspection of the NMDS ordination plot showed clusters of various states located in the Northeast, South, and West, clearly distinguished from North Dakota, Wyoming, and Oklahoma (Fig 2B). For both native bark and ambrosia beetles there was a general dissimilarity among states located in the west with those located in the other regions of the USA (Fig 2C and 2D).

### Analyses of correlates of species richness difference and species replacement

**Fig 3:**
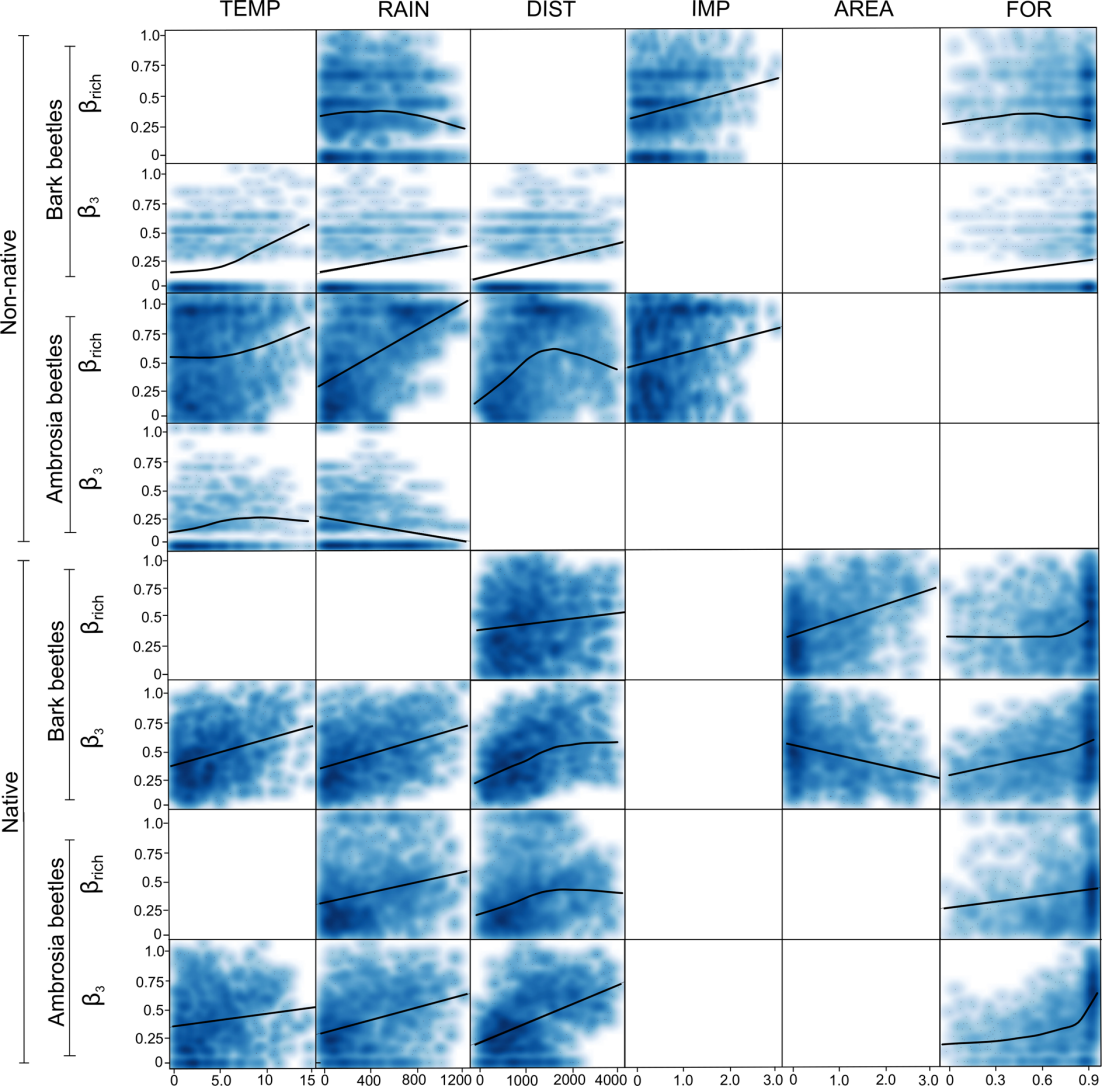
Scatterplot of the relationship for species richness difference (β_rich_) and species replacement (β_-3_) versus geographical, environmental, and human-related distances for both non-native and native bark and ambrosia beetles in the continental USA. The fitted lines are computed by “local regression” (Loess) which is a nonparametric fitting technique that does not require an a priori specification of the relationship between the dependent and independent variables [68]. The scatterplot is a smoothed shade density representation obtained through a kernel density (function ‘smoothScatter’ in graphics version 3.4.0 package in R).

For the non-native bark beetle species, we found a significant negative non-linear relationship between β_rich_ and environmental distances (rainfall and forest vegetation), and a positive linear relationship with import distances (Table 1A and Fig 3), indicating that the greater the difference between two states in terms of import volume, the greater the difference in non-native bark beetle species richness. Moreover, we found a significant and positive relationship between β_-3_ of non-native bark beetles and both geographical distance (linear) and environmental distances (non-linear for temperature, linear for rainfall and forest vegetation) (Table 1A and Fig 3), indicating that the higher the geographical distance or the difference in terms of mean annual temperature, mean annual rainfall and forest vegetation between two states, the greater the difference in the scolytine communities in terms of species composition.

**Table 1:** Results of the regression on distance matrices testing the effect of the geographical, environmental, and human-related distances on β_rich_ and β_-3_ for both non-native and native scolytines in the United States, presented separately for bark and ambrosia beetles. The significance of the slopes was evaluated by a permutation test (n = 9999). For each predictor both linear and quadratic terms were tested. R^2^ indicates the cumulative variation explained by the linear term alone or by the linear and quadratic terms together. Only significant terms are reported (P<0.01). Abbreviations: DIST: geographical distance; TEMP: difference in temperature; RAIN: difference in rainfall; FOR: difference in forest vegetation; AREA: difference in forest cover area; IMP: difference in import volume. AREA and IMP were log-transformed. See Materials and Methods for details on the above parameters.

**a) Non-native species**								
	Bark beetles				Ambrosia beetles			
	β_rich_	R^2^	β_-3_	R^2^	β_rich_	R^2^	β_-3_	R^2^
TEMP	-	-	0.050	-	0.017	-	0.051	-
TEMP^2^	-	-	0.019	0.010	0.024	0.020	-0.020	0.038
RAIN	0.007	-	0.042	0.041	0.163	0.273	-0.047	0.046
RAIN^2^	-0.028	0.020	-	-	-	-	-	-
DIST	-	-	0.056	0.072	0.133	-	-	-
DIST^2^	-	-	-	-	-0.074	0.154	-	-
IMP	0.043	0.042	-	-	0.058	0.034	-	-
IMP^2^	-	-	-	-	-	-	-	-
AREA	-	-	-	-	-	-	-	-
AREA^2^	-	-	-	-	-	-	-	-
FOR	-0.034	-	0.032	0.023	-	-	-	-
FOR^2^	-0.025	0.017	-	-	-	-	-	-
**b) Native species**								
TEMP	-	-	0.070	0.105	-	-	0.033	0.019
TEMP^2^	-	-	-	-	-	-	-	-
RAIN	-	-	0.081	0.142	0.060	0.059	0.076	0.098
RAIN^2^	-	-	-	-	-	-	-	-
DIST	0.029	0.017	0.104	-	0.068	-	0.112	0.215
DIST^2^	-	-	-0.026	0.192	-0.041	0.067	-	-
IMP	n.a.	n.a.	n.a.	n.a.	n.a.	n.a.	n.a.	n.a.
IMP^2^	n.a.	n.a.	n.a.	n.a.	n.a.	n.a.	n.a.	n.a.
AREA	0.085	0.152	-0.062	0.085	-	-	-	-
AREA^2^	-	-	-	-	-	-	-	-
FOR	0.069	-	0.098	-	0.037	0.022	0.183	-
FOR^2^	0.026	0.048	0.015	0.144	-	-	0.060	0.289

For the non-native ambrosia beetle species, we found a significant hump-shaped relationship between β_rich_ and geographical distance, a non-linear relationship with temperature distance, and a positive linear relationship between β_rich_ and rainfall and import differences (Table 1A and Fig 3), indicating that the greater the difference in terms of mean annual temperature, mean annual rainfall, and forest composition between two states, the greater will be the differences in non-native ambrosia beetle species richness. Moreover, for non-native ambrosia beetle species, we found a negative linear relationship between their β_-3_ and rainfall distances (Table 1A and Fig 3), and a non-linear relationship with temperature distances.

For the native bark beetle species, we found a significant positive relationship between β_rich_ and both geographical and forest cover area distances (Table 1B and Fig 3), indicating that the greater the geographical distance and the differences in terms of forest cover area between two states, the greater the difference in native bark beetle species richness. Furthermore, we found a non-linear relationship between β_rich_ and forest vegetation distances (Fig 3), indicating that differences in native bark beetle species richness are evident only among states that differ considerably in terms of forest vegetation. Moreover, we found a positive relationship between β_-3_ and all the environmental distances (linear for temperature and rainfall, non-linear for forest vegetation), as well as a negative linear relationship with forest cover area (Table 1B and Fig 3). These findings indicated that the greater the differences in terms of mean annual temperature, mean annual amount of rainfall, and forest composition between two states, the more the native bark beetle communities differ in terms of species composition, whereas the opposite trend occurs for forest cover area. A similar trend was found for native ambrosia beetles β_-3_ (except for forest cover area), whereas their β_rich_ was significantly and positively associated only with rainfall (linear), forest vegetation (linear) and geographical distances (non-linear) (Table 1B and Fig 3).

**Fig 4:**
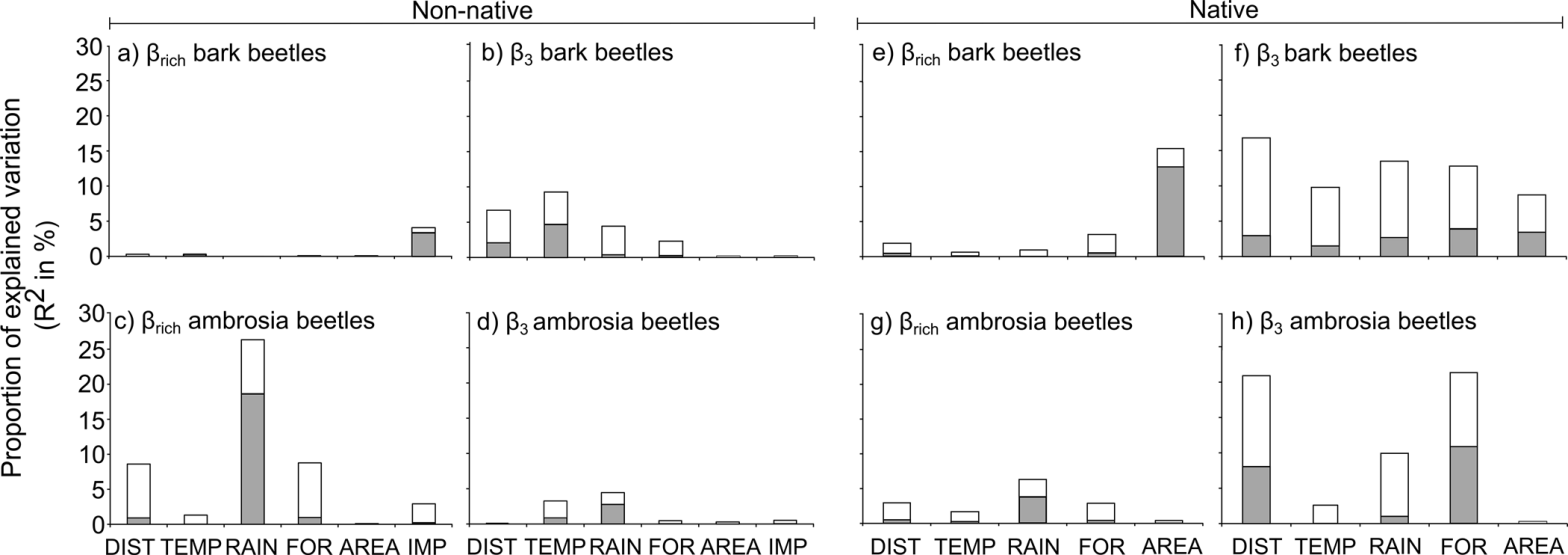
The independent (white) and shared (grey) contributions estimated from hierarchical partitioning of each explanatory variable for species richness differences and species replacement of non-native and native bark and ambrosia beetle in the USA. Abbreviations: DIST: geographical distance; TEMP: difference in temperature; RAIN: difference rainfall; FOR: difference in forest vegetation; AREA: difference in forest cover area; IMP: difference in import volume. AREA and IMP were log-transformed.

Concerning the relative importance of the correlates, the results showed that β_rich_ of non-native bark beetles was mainly explained by the differences in the volume of imported commodities between states (Fig 4A), whereas the difference in the mean annual temperature was the main explanatory variable for their β_-3_, followed by geographical distance and mean annual amount of rainfall (Fig 4B). For non-native ambrosia beetles, β_rich_ was mainly explained by differences in mean annual rainfall between states (Fig 4C), while β_-3_ was influenced by both mean annual rainfall and mean annual temperature differences (Fig 4D). Concerning native bark beetle species, β_rich_ was mainly explained by difference in forest cover area (Fig 4E), whereas the β_-3_ was strongly influenced by both environmental (differences in mean annual temperature, mean annual rainfall, and forest vegetation) and geographical distances (Fig 4F). The same correlates were the main explanatory variables for native ambrosia beetle species β_rich_ (Fig 4G), whereas their β_-3_ was mainly influenced by the geographical distance and the differences in forest vegetation and mean annual amount of rainfall (Fig 4H).

## Discussion

In the USA, when considering all established non-native tree-feeding insects, there is a notable concentration in the northeast, with decreasing numbers to the west and south [31]. This trend has been shown for different groups of invasive organisms [31], but for scolytines the mechanisms of invasion and the factors shaping their communities are still largely understudied. Our large-scale study elucidates some of these aspects, supporting the hypothesis that differential establishment and spread of bark and ambrosia beetles was likely influenced by their feeding habit and climatic factors in the new environment.

For non-native bark beetles, we found that the species pool at the state level differs more in terms of species richness than species replacement, but only the latter component showed a spatial autocorrelation, indicating a higher similarity among communities of neighboring states. This scenario may reflect a discontinuous invasion process composed of long distance jumps or multiple introduction events, supported by the lack of a spatial trend in species richness differences, and short-distance movements, supported by the similar species composition of neighboring states. Multiple introductions and human-assisted dispersal have already been identified as key processes for the establishment and spread of non-native insects throughout wide areas [33–37], and for wood-boring beetles these mechanisms are often related either to movement of imported goods and associated wood-packaging materials towards their final destinations [69] or firewood carried by visitors to recreational areas or vacation properties [70,71]. Bark beetles can, however, also spread naturally in areas surrounding the initial point of entry exploiting their ability to fly long distances [39,72], and this might help explain why states close to each other present similar communities. Analyzing in more detail the correlates of the two components of β-diversity, we found that differences in import volume mainly explained species richness differences, whereas differences in temperature mainly explained species replacement. Although the effect of import volume on the number of established bark beetles was previously highlighted by Marini et al. [8], the effect of temperature on species replacement between states was still unclear. This relationship may be influenced by environmental requirements of bark beetles [73] and their associates, such as fungi, bacteria, nematodes, and mites that can significantly influence bark beetle fitness [74,75]. For example, each fungal associate often possesses different thermal optima for growth, and variation in seasonal temperatures can influence which fungal species are ultimately vectored by dispersing bark beetles [76–78], thereby helping to shape the communities of non-native bark beetles. In general, such patterns were slightly different compared to those of native bark beetles. In particular, native species replacement followed the distance decay rule of similarity, with decreasing similarity between communities with increasing distance between them [32]. Scolytine species that are native to a given area are likely well-adapted to the local climatic conditions, thus, as the climatic conditions change, the pool of host plants and native scolytines also changes. The significant role of geographical distance and environmental factors in explaining the species replacement of native bark beetles supports this trend.

For non-native ambrosia beetles, we found that the communities differed mainly in terms of species richness. Furthermore, we found a clear spatial autocorrelation for this component, represented by gradual species richness changes with increasing geographical distance. This situation may reflect a more continuous invasion process, with the pool of non-native species arriving in the coastal areas that tends to be filtered as they spread, either naturally or assisted by humans, to internal portions of the continental USA. Such differences in species richness appeared to be primarily associated with differences in amount of rainfall between states. Ambrosia beetles are strictly dependent on their symbiotic fungi, which need certain conditions to grow and develop [79–81]. This dependency appears to limit the geographic distribution of many species of ambrosia beetles to wetter and warmer regions of the USA [8]. For example, much of the central and interior western parts of the USA are characterized by significantly lower rainfall as compared to the eastern USA [63], which appears to restrict establishment and spread of many non-native ambrosia beetles [8,80]. For native ambrosia beetles, instead, we found slightly different patterns. For both species richness differences and species replacement, there was a tendency for the values to decrease as distance between states increased. If species richness differences were mainly explained by rainfall differences, which is related to the strong dependence that ambrosia beetles have on climate [8,80], then species replacement was primarily influenced by geographical distance and differences in forest composition. Although the effect of the geographical distance could be expected [32], that of forest composition might be somewhat unexpected given that most ambrosia beetles are rather polyphagous in terms of host plants colonized [79,81]. It is reasonable to think that ambrosia beetle species that are native within a given area are more adapted to certain habitats and hosts than others, thus, as the composition and characteristics of forest stands change, the ambrosia beetles communities also change.

Our study suggests that bark and ambrosia beetles differed in their spread pattern across the invaded environment. The lower dependency that bark beetles have for strict climatic conditions may allow them to potentially colonize larger areas within the USA, and they are therefore more likely to become established in distant regions if moved by humans. Moreover, the effect of temperature on species replacement in bark beetles and their relatively high host specificity might be used to model or predict which species are likely to arrive and establish in certain areas, taking into account the most common sources of the imported goods at nearby ports. Regarding ambrosia beetles, non-native species can spread through natural or human-mediated processes, but they will typically become established only in those areas where the climatic conditions are suitable for their reproduction and development. Although ambrosia beetles are generally polyphagous, conducting studies on host specificity of selected non-native ambrosia beetles could help identify which areas of a country are most at risk of ambrosia beetle invasion. Nevertheless, it is important to remember one of the main limitations of our dataset, which is the possible difference in scolytine trapping efforts among states. For this reason, further research should be conducted when additional information becomes available. At the same time, it would be valuable to integrate our results with data collected at finer spatial scales in order to verify if the variables affecting bark and ambrosia beetle establishment and spread at smaller spatial scales match or not those highlighted in the current study. This information, integrated with a modeling approach [82], should prove useful for decision makers responsible for implementing national biosecurity strategies and monitoring programs for non-native scolytines, giving forest health managers a tool to prioritize where resources should be allocated for best intercepting and detecting alien species within their country or region of interest.

## Supporting Information

S1 Table List of non-native bark and ambrosia beetle species considered in this study. For each species the key reference reporting the first finding in the USA is indicated.
(DOC)
