# Supplementary material for: Bark and Ambrosia Beetles Show Different Invasion Patterns in the USA
Source: PLoS One. 2016 Jul 26;11(7):e0158519. doi: 10.1371/journal.pone.0158519 (PMC4961435; doi:10.1371/journal.pone.0158519)
Supplement: S1 Table — For each species the key reference reporting the first finding in the USA is indicated. (DOC) [file pone.0158519.s001.doc]

**Table S1 List of non-native bark and ambrosia beetle species considered in this study**. For each species the key reference reporting the first finding in the USA is indicated. Species are listed alphabetically.

| **Species** | **Tribe** | **References** |
| --- | --- | --- |
| ***Ambrosia beetles*** |  |  |
| *Ambrosiodmus lewisi* (Blandford) | Xyleborini | [1] |
| *Ambrosiodmus rubricollis* (Eichhoff) | Xyleborini | [2] |
| *Ambrosiophilus atratus* (Eichhoff) | Xyleborini | [3] |
| *Anisandrus dispar* (Fabricius) | Xyleborini | [4] |
| *Anisandrus maiche* Stark | Xyleborini | [5] |
| *Cnestus mutilatus* (Blandford) | Xyleborini | [6] |
| *Cyclorhipidion californicus* (Wood) | Xyleborini | [7] |
| *Cyclorhipidion pelliculosum* (Eichhoff) | Xyleborini | [3] |
| *Dryoxylon onoharaensum* (Murayama) | Dryocoetini | [8] |
| *Euwallacea fornicatus* (Eichhoff) | Xyleborini | [9] |
| *Euwallacea validus* (Eichhoff) | Xyleborini | [10] |
| *Premnobius cavipennis* Eichhoff | Premnobini | [2] |
| *Wallacellus similis* (Ferrari) | Xyleborini | [11] |
| *Xyleborinus alni* (Niisima) | Xyleborini | [12] |
| *Xyleborinus andrewesi* (Blandford) | Xyleborini | [4] |
| *Xyleborinus octiesdentatus* (Murayama) | Xyleborini | [13] |
| *Xyleborinus saxesenii* (Ratzeburg) | Xyleborini | [14] |
| *Xyleborus glabratus* Eichhoff | Xyleborini | [11] |
| *Xyleborus pfeilii* (Ratzeburg) | Xyleborini | [15] |
| *Xyleborus seriatus* Blandford | Xyleborini | [16] |
| *Xylosandrus amputatus* (Blandford) | Xyleborini | [17] |
| *Xylosandrus compactus* (Eichhoff) | Xyleborini | [9] |
| *Xylosandrus crassiusculus* (Motschulsky) | Xyleborini | [18] |
| *Xylosandrus germanus* (Blandford) | Xyleborini | [19] |
| *Trypodendron domesticum* (Linnaeus) | Xyloterini | [20] |
| ***Bark beetles*** |  |  |
| *Crypturgus pusillus* (Gyllenhal) | Crypturgini | [21] |
| *Hylastes opacus* Erichson | Hylastini | [22] |
| *Hylurgops palliatus* (Gyllenhal) | Hylastini | [23] |
| *Hylurgus ligniperda* (Fabricius) | Tomicini | [24] |
| *Hypocryphalus mangiferae* (Stebbing) | Cryphalini | [25] |
| *Hypothenemus birmanus* (Eichhoff) | Cryphalini | [25, 10] |
| *Hypothenemus brunneus* (Hopkins) | Cryphalini | [26, 10] |
| *Hypothenemus columbi* Hopkins | Cryphalini | [26, 10] |
| *Hypothenemus erectus* LeConte | Cryphalini | [27, 10] |
| *Hypothenemus javanus* (Eggers) | Cryphalini | [28, 10] |
| *Orthotomicus erosus* (Wollaston) | Ipini | [29] |
| *Phloeosinus armatus* Reitter | Phloeosinini | [22] |
| *Pityogenes bidentatus* (Herbst) | Ipini | [30] |
| *Scolytus mali* (Bechstein) | Scolytini | [31] |
| *Scolytus multistriatus* (Marsham) | Scolytini | [32] |
| *Scolytus rugulosus* (Muller) | Scolytini | [33] |
| *Scolytus schevyrewi* Semenov | Scolytini | [34] |
| *Tomicus piniperda* (Linnaeus) | Tomicini | [35] |

**References**

[1] Hoebeke ER. An Asian ambrosia beetle, *Ambrosiodmus lewisi*, new to North America (Coleoptera: Scolytidae). Proc Entomol Soc Wash. 1991; 93: 420–424.

[2] Bright DE. Review of the tribe Xyleborini in America north of Mexico (Coleoptera: Scolytidae). Can Entomol. 1968; 100: 1288–1323.

[3] Atkinson TH, Rabaglia RJ, Bright DE. Newly detected exotic species of *Xyleborus* (Coleoptera: Scolytidae) with a revised key to species in eastern North America. 1990. Can Entomol. 1991; 122: 93–104.

[4] Peck WD. On the insects which destroy young branches of the pear tree, and the leading shoot of the Weymouth pine. Mass Agric Rep.1817; 4: 205–211.

[5] Rabaglia RJ, Vandenberg NJ, Acciavatti RE. First records of *Anisandrus* *maiche* Stark (Coleoptera: Curculionidae: Scolytinae) in North America. Zootaxa. 2009; 2137: 23–28.

[6] Schiefer TL, Bright DE. *Xylosandrus mutilatus* (Blandford), an exotic ambrosia beetle (Coleoptera: Curculionidae: Scolytinae: Xyleborini) new to North America. Coleopt Bull. 2004; 58: 431–438

[7] Wood SL. New synonymy and new species of American bark beetles (Coleoptera: Scolytidae), Part II. Great Basin Nat*.* 1975; 35: 391–401.

[8] Bright DE, Rabaglia RJ. *Dryoxylon*, a new genus for *Xyleborus onoharaensis* Murayama, recently established in the southeastern United States (Coleoptera: Scolytidae). Coleopt Bull. 1999; 53: 333–337.

[9] Thomas MC. Two Asian ambrosia beetles recently established in Florida (Curculionidae: Scolytinae). 2004. Available: [www.freshfromflorida.com/pi/enpp/ento/twonewxyleborines.html](http://www.freshfromflorida.com/pi/enpp/ento/twonewxyleborines.html).

[10] Wood SL. The bark and ambrosia beetles of North and Central America (Coleoptera: Scolytidae), a taxonomic monograph. Great Basin Nat Mem. 1982; 6: 1–1359.

[11] Rabaglia RJ, Dole SA, Cognato AI. Review of American Xyleborina (Coleoptera: Curculionidae: Scolytinae) occurring North of Mexico, with an illustrated key. Ann Entomol Soc Am*.* 2006; 99: 1034–1056.

[12] Mudge AD, LaBonte JR, Johnson KJR, LaGasa EH. Exotic woodboring Coleoptera (Micromalthidae, Scolytidae) and Hymenoptera (Xiphydriidae) new to Oregon and Washington. Proc Entomol Soc Wash. 2001; 103: 1011–1019.

[13] Rabaglia RJ, Knížek M, Johnson W. First records of *Xyleborinus octiesdentatus* (Murayama) (Coleoptera, Curculionidae, Scolytinae) from North America. ZooKeys. 2010; 56: 219–226.

[14] Hopkins AD. Classification of the Cryphalinae, with descriptions of new genera and species. US Department of Agriculture Report. 1915; 99: Washington, DC.

[15] Vandenberg NJ, Rabaglia RJ, Bright DE. New records of two *Xyleborus* (Coleoptera: Scolytidae) in North America. Proc Entomol Soc Wash. 2000; 102: 62–68.

[16] Hoebeke ER, Rabaglia RJ. *Xyleborus seriatus* Blandford (Coleoptera: Curculionidae: Scolytinae), an Asian ambrosia beetle new to North America. Proc Entomol Soc Wash. 2008; 110: 470–476.

[17] Cognato AI, O’Donnell R, Rabaglia RJ. An Asian ambrosia beetle, *Xylosandrus* *amputatus* (Blandford) (Curculionidae: Scolytinae: Xyleborini), discovered in Florida, USA. Coleopt Bull. 2011; 65: 43–45.

[18] Anderson DM. First record of *Xyleborus semiopacus* in the continental United States (Coleoptera: Scolytidae). US Department of Agriculture, Cooperative Economic Insect Report. 1974; 24: 863–864.

[19] Felt EP. A new pest in green house grown grape stems. J Econ Entomol. 1932; 25: 418.

[20] NAPIS (National Agricultural Pest Information System) Pest Tracker. 2008. Available: http://pest.ceris.purdue.edu/searchpest. php?selectName=INBQRCA

[21] LeConte JL. Synopsis of the Scolytidae of America north of Mexico. Am Entomol Soc Trans. 1868; 2: 150–178.

[22] Wood SL. Nomenclatural changes and new species in Platypodidae and Scolytidae (Coleoptera), Part II. Great Basin Nat. 1992; 52: 78–88.

[23] Hoebeke ER, Acciavatti RE. *Hylurgops palliatus* (Gyllenhal), an Eurasian bark beetle new to North America (Coleoptera: Curculionidae: Scolytinae). Proc Entomol Soc Wash. 2006; 108: 267–273.

[24] Hoebeke ER. *Hylurgus ligniperda*: a new exotic pine bark beetle in the United States. Newsletter of the Michigan Entomological Society*.* 2001; 46: 1–2.

[25] Wood SL. A revision of North American Cryphalini (Scolytidae: Coleoptera). Univ Kans Sci Bull*.* 1954; 36: 959–1089.

[26] Hopkins AD. Classification of the Cryphalinae, with descriptions of new genera and species. US Department of Agriculture Report. 1915; 99.

[27] LeConte JL. Family IX: Scolytidae. Proc Am Phil Soc. 1876; 15: 341–390.

[28] Wood SL. Introduced and exported American Scolytidae (Coleoptera). Great Basin Nat. 1977; 37: 67–74.

[29] Lee JC, Smith SL, Seybold SJ. Mediterranean pine engraver. US Department of Agriculture, Forest Service, State and Private Forestry, Pacific Southwest Region, 2005. Pest Alert R5-PR-016, Susanville, California.

[30] Hoebeke ER. *Pityogenes bidentatus* (Herbst), a European bark beetle new to North America (Coleoptera: Scolytidae). J N Y Entomol Soc. 1989; 97: 305–308.

[31] LeConte JL. Synopsis of the Scolytidae of America north of Mexico. T Am Entomol Soc. 1868; 2: 150–178.

[32] Chapman JW. The introduction of a European Scolytid (the smaller elm bark beetle, *Scolytus multistriatus* Marsh.) into Massachusetts. Psyche. 1910; 17: 63–68.

[33] Chittenden FH. Fruit-tree bark beetle (*Scolytus rugulosus* Ratz.). US Department of Agriculture, Division of Entomology, 1898. Series 2, Circular 29, Washington, DC.

[34] Negrón JF, Witcosky JJ, Cain RJ, LaBonte JR, Duerr DA, McElwey SJ, Lee JC, et al. The banded elm bark beetle: a new threat to elms in North America. Am Entomol. 2005; 51: 84–94.

[35] Haack RA, Poland TM. Evolving management strategies for a recently discovered exotic forest pest: the pine shoot beetle, *Tomicus piniperda* (Coleoptera). Biol Invasions. 2001; 3: 307–322.
